# Low Cost MR Compatible Haptic Stimulation with Application to fMRI Neurofeedback

**DOI:** 10.3390/brainsci10110790

**Published:** 2020-10-28

**Authors:** Kymberly D. Young, Nicole Prause, Sarah Lazzaro, Greg J. Siegle

**Affiliations:** 1 Department of Psychiatry, University of Pittsburgh School of Medicine, Pittsburgh, PA 15213, USA; lazzarosk2@upmc.edu (S.L.); gsiegle@pitt.edu (G.J.S.); 2 Liberos, Los Angeles, CA 95812, USA; nicole.prause@gmail.com

**Keywords:** fMRI neurofeedback, haptic, vibration, amygdala

## Abstract

The most common feedback displays in the fMRI environment are visual, e.g., in which participants try to increase or decrease the level of a thermometer. However, haptic feedback is increasingly valued in computer interaction tasks, particularly for real-time fMRI feedback. fMRI-neurofeedback is a clinical intervention that has not yet taken advantage of this trend. Here we describe a low-cost, user-friendly, MR-compatible system that can provide graded haptic vibrotactile stimulation in an initial application to fMRI neurofeedback. We also present a feasibility demonstration showing that we could successfully set up the system and obtain data in the context of a neurofeedback paradigm. We conclude that vibrotactile stimulation using this low-cost system is a viable method of feedback presentation, and encourage neurofeedback researchers to incorporate this type of feedback into their studies.

## 1. Introduction

Haptic, or tactile, feedback is an increasingly common modality for interacting with computer systems given its potential to increase learning [1,2], particularly in virtual reality clinical contexts [3], to provide analogs of real-world experiences [4], and to provide physiologically reactive stimulation [5]. For all of these reasons, haptic feedback systems have been implemented for mechanistic studies using neuroimaging [6,7,8,9], particularly including devices that include vibrotactile stimulation [9,10,11,12,13]. Neurofeedback, in which individuals learn to manipulate brain function, has specifically been shown to benefit from such vibrotactile haptic feedback [14,15,16,17]; biofeedback studies more generally have also shown the benefits of this vibratory modality [18]. There are many neuroimaging methods for which haptic feedback could be applicable [19]; here we focus on fMRI, in which, despite a number of proofs-of-concept, vibrotactile stimulation during fMRI is not yet common [20]. Here, we consider the application of vibrotactile stimulation specifically for fMRI neurofeedback as an example domain in which to overcome the commonly perceived obstacles to its implementation.

Real-time functional magnetic resonance imaging neurofeedback (rtfMRI-nf) is becoming a commonly used tool to manipulate hemodynamic activity, with the goals of both better understanding brain–behavior/cognition relationships as well as creating new interventions for clinical illnesses [20,21,22]. In a typical neurofeedback setup, participants are instructed to increase or decrease a feedback signal presented to them. This signal is generated by extracting images from the MR scanner, analyzing activity or connectivity in specific brain regions online, and quantifying this activity to a simple feedback signal (most commonly a single value). The most commonly used feedback modality is a visual display [22]. While a large variety of displays have been used (including computer games, brain activation maps and social reinforcement; see [22]), the most commonly used visual display is a thermometer.

We have conducted several studies with the goal of training participants to increase hemodynamic activity in their amygdala while recalling positive autobiographical memories [23,24,25]. These studies have demonstrated that both healthy and depressed participants can increase their amygdala hemodynamic response during positive memory recall, and this has large effects on depressive symptoms and processing biases [25,26]. While participants are generally successful in the neurofeedback task, many comment that they wished they could have closed their eyes during the feedback so as to more fully immerse themselves in the memories. Haptic feedback would solve this issue, and indeed would be especially useful for paradigms that involve savoring or rumination. Haptic feedback also offers a method for probing touch senses, such as c-afferent fibers (rather than just visual), and offers an alternative for those who are visually-impaired.

Haptic feedback may remain rare in fMRI studies, particularly fMRI neurofeedback, because fMRI-compatible haptic systems are often high-cost or are perceived to be too niche or complicated for easy implementation by non-engineers. For example, many of the primary publications cited above use custom systems and are published in engineering journals. The publicly available systems tend to be much more complex and sensitive than what is needed for a simple vibrotactile stimulation or neurofeedback, wherein the primary requirement is to be able to sense different amplitudes or frequencies of stimulation. Here, our goals are to describe how a non-engineer can build a simple fMRI-compatible haptic feedback system for under USD 150 in approximately 2 h, and to show the feasibility of using this system in the context of a real-time fMRI neurofeedback protocol in a case series with *N* = 3. Our specific questions included (1) whether, in a small sample, neurofeedback effects could be detected with haptic as well as visual stimulation (i.e., non-inferiority), (2) whether the effect of the haptic stimulation, in the absence of neurofeedback, would likely occlude other signals in areas of interest (here, the amygdala and intraparietal sulcus (IPS)), and (3) whether the effect of haptic stimulation would be detectable in regions associated with responses to somatic stimuli (insula and somatosensory cortex) to validate the interpretation of question 2. These questions are important to answer given the wealth of data suggesting that haptic neurofeedback could offer advantages that visual feedback cannot, e.g., allowing participants to close their eyes in the scanner and having inherent primary hedonic features.

## 2. Materials and Methods

### 2.1. Haptic Setup for MRI

The goal for the MRI haptic setup is to stay within the budget and capabilities of a technically-interested undergraduate who has no special electrical skills. Typical MRI software geared to either provoke different levels of reactivity or for neurofeedback provides a numeric output, based on some neural activity, that is commonly used to create visual feedback. This value can also be scaled to operate haptic feedback representing the strength of desired haptic stimulation (e.g., higher when activity is higher). Specifically, that number can be translated into vibration. In addition, metals cannot safely exist in the scanner environment. To address this constraint, this vibration is generated outside the scanner, and a rigid form transmits the vibration into the scanner bore. The system thus has three parts: a controller (Arduino microcontroller), a vibrating element (vibrating motor), and a way to get the vibration from outside the scanner to inside the scanner (PVC tubing). Each of these elements is described below. Supplementary Materials Figure S1 contains our full parts list with information about at least one source from which each piece can be ordered.

**Figure 1:**
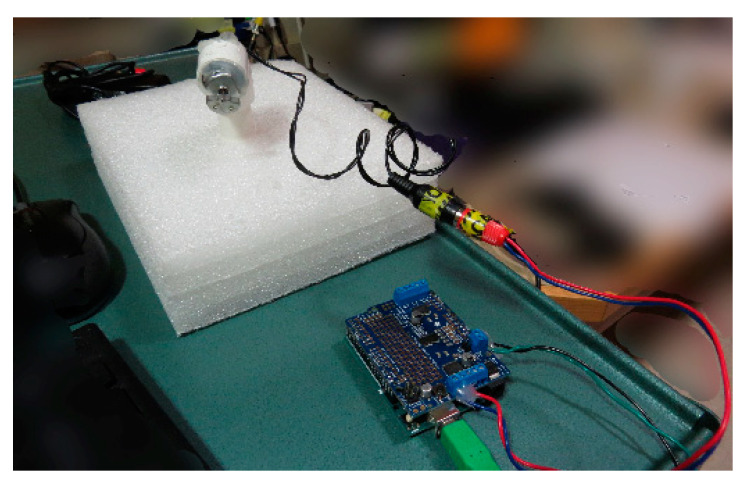
Equipment to produce vibrational stimuli—as assembled. Computer is connected, via USB, to an Arduino Uno with an Adafruit Motor shield (bottom). The motor outputs are connected to an RCA jack for ease of disassembly (center), which is connected, via another RCA jack (center), to a motor, which is inserted and glued into a PVC tube (top left) in the control room. This setup is sufficient to produce vibrations through the length of the PVC tube when they are triggered by the computer.

**Figure 2:**
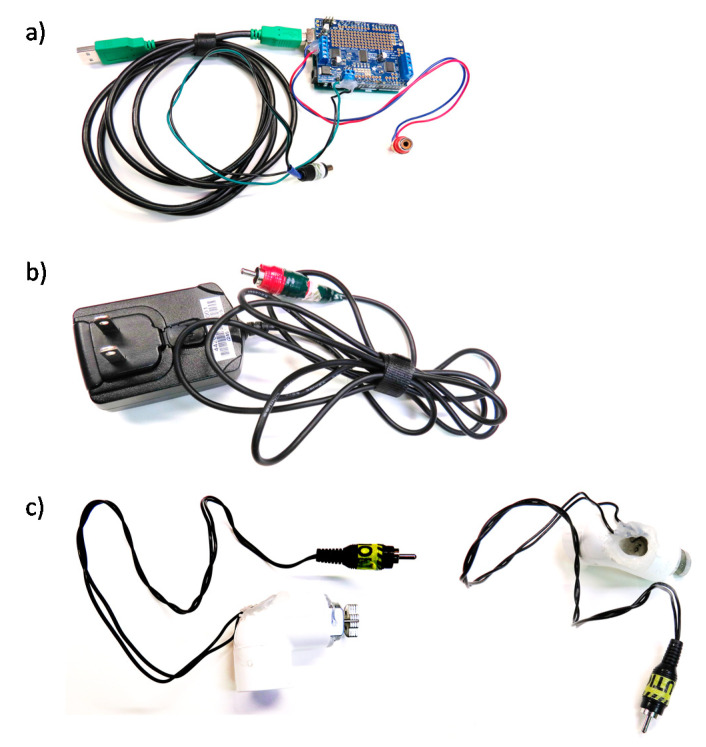
Equipment to produce vibrational stimuli—in parts. (**a**) Computer is connected, via USB, to an Arduino Uno with an Adafruit Motor shield. (**b**) The motor shield is powered externally by a 6V 1A AC to DC power source. We added RCA jacks to the power source and Arduino (in A) for easy assembly/disassembly. (**c**) The motor outputs of the Arduino are connected, via RCA jacks, to a 3V motor, which is inserted and glued into a PVC tube (Side and top views shown).

Controller (USD 50)—Arduino Uno (~USD 25; Arduino LLC, Boston, MA, USA) and an Adafruitmotor shield (~USD 20; Arduino LLC, Boston, MA, USA) (Figure 1, which shows our vibration-controller setup as we assembled it). We have used both the original Arduino Motor Shield (which one can buy pre-assembled) and the Adafruit Motor Shield v2. The Adafruit may be supported for longer, but requires some soldering for connections to be robust. In addition, wires will need to be screwed into the Arduino, which could go right to what you use to generate the vibration signal. Alternatively, and easier to take apart, the wires from the Adafruit can be converted to end with a Radio Corporation of American (RCA) jack that is commonly included in consumer audio equipment. Such pre-assembled RCA to screw terminal adapters are inexpensive (~USD 7) and require no soldering (Figure 2a). The motor shield also requires a power source. We use an external 5 volt 2 Amp direct current power supply (~USD 8). These standard plugs can be purchased, then the ends cut off, wires stripped, and wires run into the motor shield’s power input receptacles (Figure 2b). To preserve robustness in the scanning environment, we hot glued over all of the screw terminals once the relevant wires were connected.

Software—The Arduino is a microcontroller, which must be loaded with a program that allows it to be controlled by a computer. Software that uploads firmware from any computer to the Arduino is freely available (Arduino 1.8.13, Arduino, Boston, MA, USA). Examples of motor control programs to upload are numerous and freely available. We have provided Matlab software (Matlab R2018b, Mathworks, Natick, MA, USA) for neurofeedback control, which interfaces with the Turbo-BrainVoyager™ neurofeedback module (Turbo Brain Voyager 4.0, Brain Innovations, Maastricht, The Netherlands), and which uses the default Arduino control software for Matlab, at https://www.mathworks.com/matlabcentral/fileexchange/74339-haptic-feedback-for-turbo-brainvoyager. This software assumes that Turbo-BrainVoyager™ creates a file representing the level of activity in a region of interest at each repetition time (TR) within a known range. The software continuously polls for the existence of such a file, and when a new file is found, generates a vibration of corresponding intensity. Open source options for neurofeedback software, for example Open NFT, could also be used. GNU Octave (GNU 5.2.0, GNU Operating System, Boston, MA, USA) could be used as an open source alternative to Matlab.

Vibrating Element (USD 30)—Any 3-5 volt DC vibrating motor should yield sufficient vibration to be felt at the scanner bore. We used a uxcell 5 volt DC, 3200 revolutions per minute motor (USD 6). From this motor we connected its wires (solder or hot glue can connect these) to the RCA female plug (see above). We mounted the motor and wires inside a piece of 1” pvc through which we drilled a hole for the wires, and hot glued the motor in place, leaving sufficient unglued area for air circulation to account for heating in the motor (Figure 2c).

**Figure 3:**
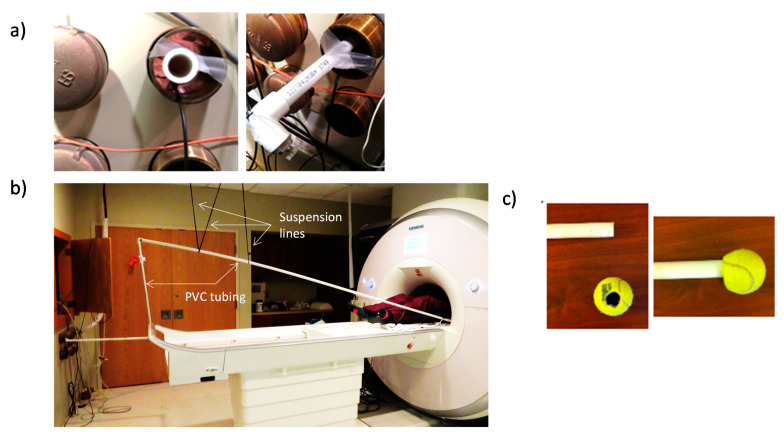
(**a**) PVC tubing suspended within the waveguide with medical tape so as not to touch the waveguide’s edges. (**b**) Extension of PVC tube from the control room to the scanner bore showing the paracord from the scanning room ceiling used to suspend the tubing; (**c**) optional tennis ball for subject to hold.

Tube to transfer vibration to the subject (USD 30)—To get from a typical MRI control room, through the waveguide, to our scanner bore, we use approximately 30 feet of 1 inch PVC tube (USD 15). We included angle couplings to navigate around the room’s objects as necessary. The length and topology for a given scanner will depend on measurements specific to the users’ scanning environment. If the PVC touches the waveguide, some of the vibration being transmitted will be lost/dampened. To reduce the loss of vibratory energy, we suspend the PVC through the waveguide from the scanner room ceiling (Figure 3a) using string, paracord, or medical tape (USD < 10) (Figure 3b). The participant may receive the vibration by adding any number of coverings or endpoints to the PVC tubing, e.g., allowing a participant to hold a tennis ball with a hole into which the PVC tube is inserted (Figure 3c).

Design alternatives: We have explored many design alternatives to the current system. Prause et al. (2012) [13] used a system with an air compressor, tubing and an air-powered imbalanced turbine in lieu of the vibrating motor and PVC [12]. This system worked well for us, but was much louder and had less power. For applications which do not require graded vibration, but rather can use a simple on–off switch, a simpler approach can work to achieve even stronger haptic feedback. In lieu of the motor shield and motor, the Arduino’s digital outputs and ground can be connected to a controllable outlet power relay (IoT Power Relay available from AdaFruit for USD 25), into which many commercially available vibrating devices can be attached (e.g., personal massager with a reducing coupler for the head or a sheet pad sander, for which copper brackets can be screwed into the sander plate and around the PVC to tightly and stably connect to the PVC tube (Figure S2)). Arduino code for these applications is available from GJS by request.

### 2.2. Procedure

To assess the feasibility of the system for fMRI neurofeedback, three medically healthy individuals performed neurofeedback with alternating runs of vibration off and on (Subject 1, F, age 36; Subject 2, F, age 23; Subject 3, M, age 34). Written informed consent was obtained from the participants. The study was approved by the University of Pittsburgh Institutional Review Board (Identification Code STUDY19050176) and carried out in accordance with the Declaration of Helsinki for experiments involving humans. We used the commercially available Turbo-BrainVoyager™ software for real-time imaging and processing. The rtfMRI-nf procedure consisted of five fMRI runs each lasting 8 min and 40 s, a baseline run in which no neurofeedback information was provided, and four training runs. During training runs 1 and 3, no vibration was provided and the standard thermometer was used, while in runs 2 and 4 the thermometer was visible and vibration feedback was also provided. This design has been published previously and fully described elsewhere [22,23]. Briefly, all runs consisted of alternating blocks of Rest (5 40 s blocks), Count Backwards (4 40 s blocks of counting backwards from 300 by an integer), and Happy/Regulate (4 40 s blocks). During the Happy condition, participants were instructed to silently recall and contemplate positive autobiographical memories while also attempting to increase the level of the thermometer and/or the strength of the vibration felt. An empty thermometer was displayed during the Count and Rest conditions and no vibration was felt.

The neurofeedback signal for each Happy block was computed as the fMRI percent signal change relative to the average fMRI signal for the preceding Rest block. This was provided as output over every 2 s window during happy recall and presented to the participant both visually (thermometer) and haptically (vibration) to their right hand. To reduce fluctuations due to noise in the fMRI signal, the thermometer level and strength of vibration was computed at every time point as a moving average of the current and two preceding values. These percent signal change values obtained during neurofeedback were averaged over each run and used as a performance measure (the signal the participants received). These values were used to compare amygdala activity during vibration on vs. off, as we were interested in how vibration affected the signal being trained. To examine differences between the vibration on and off conditions, we performed an area under the curve (AUC) test of the mean of the vibration on condition (minus the preceding rest run) versus the no vibration condition (minus the preceding rest run).

The amygdala region-of-interest was defined as a sphere of 7 mm radius centered at −21, −5, −16 in the stereotaxic array of Talairach and Tournoux, and was transformed to the EPI image space using each subject’s high-resolution MPRAGE structural data. The resulting region-of-interest in the EPI space contained approximately 140 voxels. We performed a visual inspection of the regions-of-interest prior to the start of neurofeedback. No adjustments were performed as a result of visual inspection.

After the feedback task was complete, the participants received variable vibration that was not associated with their own amygdala activity. Specifically, each received the neurofeedback vibration of another participant during an 8 min 40 s resting state run, during which the instructions were to simply relax and not think of anything in particular. This was done to examine whether the amygdala was activated by vibration in the absence of a task. This yoked sham neurofeedback signal was created from the first training run of a female subject from another study with depression who completed our standard fMRI-neurofeedback paradigm. The first training run was selected so as to have the most variance in the vibration, as this was the run from that study wherein the participants were just beginning to learn how to effectively control the signal.

fMRI was conducted on a 3 T Siemens Prisma scanner with a 64-channel head coil. A single-shot gradient-recalled EPI sequence with GeneRalized Autocalibrating Partial Parallel Acquisition (GRAPPA) was employed for fMRI. The following EPI imaging parameters were used: FOV/slice = 260/2.9 mm, interleaved slices per volume = 34, slice thickness = 2.9 mm, repetition/echo time TR/TE = 2000/30 ms, GRAPPA acceleration factor = 2 in the phase encoding (anterior-posterior) direction, flip angle = 90°, number of volumes = 263, voxel size = 2 × 2 × 2.9 mm. A T1-weighted magnetization-prepared rapid gradient-echo (MPRAGE) sequence with GRAPPA was used to provide an anatomical reference for the fMRI analysis. It had the following parameters: FOV = 256 mm, slices per slab = 208, slice thickness = 0.80 mm, voxel size = 0.8 × 0.8 × 0.8 mm TR/TE = 2400/2.24 ms, GRAPPA acceleration factor = 2, flip angle = 8°.

fMRI analysis for the resting state data was performed using AFNI (http://afni.nimh.nih.gov/afni). The single-subject analysis steps consisted of slice timing correction, within-subject realignment, coregistration between anatomical and functional images, spatial normalization to the stereotaxic array of Talairach and Tournoux, spatial smoothing (Gaussian kernel, 4 mm full width at half maximum), and finally the voxel time series were low pass filtered (cutoff 0.10 Hz).

Standard general linear model (GLM) analysis was applied with the following regressors included in the GLM model: two block stimulus conditions for the vibration analysis (on and off), six motion parameters as nuisance covariates to take into account possible artifacts caused by head motion, and five polynomial terms for modeling the baseline. The regressors were convolved with the canonical hemodynamic response function provided with Analysis of Functional NeuroImages (AFNI, Washington, DC, USA). The hemodynamic response estimates (GLM ß coefficients) were computed for each voxel within the amygdala, intraparietal sulcus, insula, and postcentral gyrus regions of interest (ROIs) using the 3dDeconvolve AFNI program and then converted to percent signal changes for vibration on versus off. The voxel-wise percent signal change data were averaged within each ROI.

## 3. Results

### 3.1. Q1: Can Neurofeedback Effects Be Detected with Haptic Stimulation? Analysis of Amygdala Reactivity with vs. without Haptic Feedback

**Figure 4:**
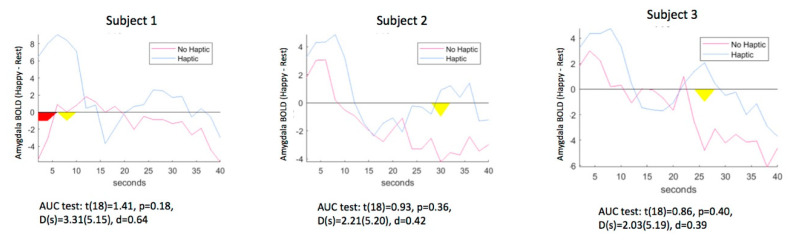
Time course of amygdala activity during neurofeedback training versus the preceding rest period with vibration on and off for each participant. Red area = significant difference at *p* < 0.05; Yellow area = significant difference at *p* < 0.10.

Table 1 shows the average amygdala values calculated on-line by Turbo-BrainVoyager™ for the Happy–Rest condition during each run and Figure 4 shows the AUC for each subject. For each participant, the observed amygdala feedback signal was higher with either type of feedback compared to the baseline (visual vs. baseline *t*(2) = 3.62, *p* = 0.06, d = 2.69; haptic vs. baseline (*t*(2) = 5.91, *p* = 0.03, d = 3.82). There was a moderate effect size for the effect of vibration, compared to visual stimulation only, across participants (d = 0.44) that was non-significant due to the small sample (*t*(2) = 0.54, *p* = 0.62).

### 3.2. Q2: Does Haptic Stimuliation Occlude Effects of Interest? Resting BOLD Response with vs. without Haptic Stimulation in Neurofeedback Regions for Which Detection of Haptic Stimuliation Would Be Problamatic (Amygdala, Intraparietal Sulcus)

Amygdala Activity: Table 2 shows the average amygdala values for haptic on–haptic off during an 8 min 40 s eyes open resting state run. The percent signal change between the two conditions was very small (one-sample *t*-test comparing mean to 0 change; mean = 0.005; *t*(2) = 0.31, *p* = 0.78) and was not in a consistent direction, and the difference between on and off showed a very small effect size (d = 0.01).

Intraparietal Sulcus Activity: We also examined the BOLD response in the control region we used in our other neurofeedback experiments, which was the left horizontal segment of the intraparietal sulcus (defined as a 7 mm sphere centered at (−42, −48, 48) in the stereotaxic array of Talairach and Tournoux). As can be seen in Table 2, the difference between the two conditions was very small (one-sample *t*-test comparing mean to 0 change; mean = −0.0004; *t* = 0.22 *p* = 0.98), not in a consistent direction, and had a very small effect size (d = 0.001).

### 3.3. Q3: Are Effects of Haptic Stimuliation Detecable? Resting BOLD Response with vs. without Haptic Stimulation in Regions Where Detection of Haptic Stimuliation Is Expected (Insula, Somatosensory Cortex)

Insula and Somatosensory Activity: We examined the BOLD response in two regions in which we expected to see greater activity when vibration was on versus off—the insula and the somatosensory cortex (postcentral gyrus). As can be seen in Table 2, in both regions bilaterally, there was increased activity when vibration was on relative to when it was off (*t*s > 9.47, *p*s < 0.001, ds > 6.86).

## 4. Discussion

We have responded to multiple theoretical papers suggesting that haptic fMRI neurofeedback could be of interest in terms of constructing a low-cost, reproducible, portable system for providing haptic feedback during rtfMRI-nf training. We made use of relatively inexpensive components that are available to consumers. The produced haptic stimulation—here vibration—has sufficient displacement and magnitude to be strongly felt while allowing for gradations indicating the amount of hemodynamic activity. The total cost was USD 130.

These feasibility data indicate that participants can increase their amygdala signal during positive autobiographical memory recall relative to a rest baseline to a similar extent when vibratory feedback is provided as when visual feedback alone is provided. Future studies should examine whether neurofeedback performance is superior with haptic relative to visual feedback. Furthermore, vibration alone during rest did not change the activity in the regions of interest for our neurofeedback studies, but did change activity in the regions we would expect to be responsive to vibration (insula and somatosensory cortex). This suggests that vibratory feedback is appropriate for neurofeedback studies targeting regions involved in emotion regulation, but caution should be used when the target is a region that is also sensitive to interoceptive signals, such as the insula, as it is possible that the effects of vibration could interfere with neurofeedback learning.

This work is a first step in bringing haptic feedback to rt-fMRI. Haptic feedback is likely to vary in its effect on results. In particular, different patterns and locations of vibration are well known to be experienced as emotionally positive or negative [27], to have different neural correlates, e.g., [9], and to have different physiological effects (e.g., whereas body vibration in the 6–10 Hz range is associated with increased indicators of sympathetic tone [28], vibration in the 89 Hz range on the face is associated with increased parasympathetic tone [29]). Thus, research establishing parameters for how haptic neurofeedback is used is a prudent next-step and a future direction of our research. Of course, larger studies in non-biased samples with a randomized block design are needed. The importance of the current work is to establish that these next steps are worth taking.

## 5. Conclusions

In conclusion, we have demonstrated that vibration during fMRI neurofeedback is well motivated, affordable, easy to implement, feasible to use, and is likely to yield interpretable results which are at least comparable to current neurofeedback methods, allow participants to close their eyes, and are not compromised by producing spurious brain activity. In our future rtfMRI neurofeedback studies, we plan to incorporate this haptic feedback.

## Figures and Tables

**Table 1:** Amygdala Hemodynamic Activity during Neurofeedback during Vibration vs. Visual Feedback.

	Subject 1	Subject 2	Subject 3	Overall Mean
Baseline	−0.41	−0.10	−0.18	−0.23
Visual Run 1	0.26	0.58	0.03	0.29
Haptic Run 1	0.33	0.51	0.22	0.35
Visual Run 2	0.55	0.76	0.17	0.49
Haptic Run 2	0.61	0.99	0.40	0.67
Mean Visual All Runs	0.41	0.67	0.10	0.39
Mean Haptic All Runs	0.47	0.75	0.31	0.51

**Table 2:** Regional Hemodynamic Activity during Rest with Vibration On vs. Off.

	% Signal Change Vibration On—Off					
	Left Amygdala	Left Intraparietal Sulcus	Left Insula	Right Insula	Left Somatosensory Cortex	Right Somatosensory Cortex
Subject 1	0.01	0.02	0.34	0.19	0.30	0.29
Subject 2	−0.01	−0.01	0.28	0.27	0.36	0.36
Subject 3	0.01	−0.01	0.19	0.20	0.26	0.29

## Supplementary Materials

The following are available online at https://www.mdpi.com/2076-3425/10/11/790/s1, Figure S1: Haptic Part List: full parts list with information about at least once source from which each piece can be ordered; Figure S2: Design Alternatives for the Haptic Setup.

## References

[1] Fleury M., Lioi G., Barillot C., Lecuyer A. (2020). A survey on the Use of Haptic Feedback for Brain-Computer Interfaces and Neurofeedback. Front. Neurosci..

[2] Kosmyna N., Maes P. (2019). AttentivU: An EEG-based closed loop biofeedback system for real-time monitoring and improvement of engagement for personalized learning. Sensors.

[3] Kim Y.I., Jung S.Y., Min S., Seol E., Seo S., Hur J.W., Jung D., Lee H.J., Lee S., Kim G.J. (2019). Visuo-Haptic-Based Multimodal Feedback Virtual Reality Solution to Improve Anxiety Symptoms: A Proof-of-Concept Study. Psychiatry Investig..

[4] Overtoom E.M., Horeman T., Jansen F.W., Dankelman J., Schreuder H.W.R. (2019). Haptic Feedback, Force Feedback, and Force-Sensing in Simulation Training for Laparoscopy: A Systematic Overview. J. Surg. Educ..

[5] Prause N., Siegle G.J., Deblieck C., Wu A., Iacoboni M. (2016). EEG to Primary Rewards: Predictive Utility and Malleability by Brain Stimulation. PLoS ONE.

[6] Menon S., Zhu J., Goyal D., Khatib O. (2017). Haptic fMRI: Reliability and performance of electromagnetic haptic interfaces for motion and force neuroimaging experiments. Conf. Proc. IEEE Eng. Med. Biol. Soc..

[7] Maddahi Y., Zareinia K., Tomanek B., Sutherland G.R. (2018). Challenges in developing a magnetic resonance-compatible haptic hand-controller for neurosurgical training. Proc. Inst. Mech. Eng. H J. Eng. Med..

[8] Vigaru B., Sulzer J., Gassert R. (2015). Design and Evaluation of a Cable-Driven fMRI-Compatible Haptic Interface to Investigate Precision Grip Control. IEEE Trans. Haptics.

[9] Kim J., Chung Y.G., Chung S.-C., Bülthoff H.H., Kim S.-P. (2016). Neural Categorization of Vibrotactile Frequency in Flutter and Vibration Stimulations: An fMRI Study. IEEE Trans. Haptics.

[10] Puckett A.M., Bollmann S., Junday K., Barth M., Cunnington R. (2020). Bayesian population receptive field modeling in human somatosensory cortex. NeuroImage.

[11] Malone P.S., Eberhardt S.P., Wimmer K., Sprouse C., Klein R., Glomb K., Scholl C.A., Bokeria L., Cho P., Deco G. (2019). Neural mechanisms of vibrotactile categorization. Hum. Brain Mapp..

[12] Wu Y.-H., Velenosi L.A., Schröder P., Ludwig S., Blankenburg F. (2018). Decoding vibrotactile choice independent of stimulus order and saccade selection during sequential comparisons. Hum. Brain Mapp..

[13] Prause N., Roberts V., Legarreta M., Cox L.M.R. (2012). Clinical and research concerns with vibratory stimulation: A review and pilot study of common stimulation devices. Sex. Relatsh. Ther..

[14] Wang Z., Zhou Y., Chen L., Gu B., Liu S., Xu M., Qi H., He F., Ming N. (2019). A BCI based visual-haptic neurofeedback training improves cortical activations and classification performance during motor imagery. J. Neural Eng..

[15] Vukelić M., Gharabaghi A. (2015). Oscillatory entrainment of the motor cortical network during motor imagery is modulated by the feedback modality. NeuroImage.

[16] Rossi-Izquierdo M., Ernst A., Soto-Varela A., Santos-Pérez S., Faraldo-García A., Sesar-Ignacio Á., Basta D. (2013). Vibrotactile neurofeedback balance training in patients with Parkinson’s disease: Reducing the number of falls. Gait Posture.

[17] Basta D., Rossi-Izquierdo M., Soto-Varela A., Greters M.E., Bittar R.S., Steinhagen-Thiessen E., Eckardt R., Harada T., Goto F., Ogawa K. (2011). Efficacy of a vibrotactile neurofeedback training in stance and gait conditions for the treatment of balance deficits: A double-blind, placebo-controlled multicenter study. Otol. Neurotol..

[18] Gonçalves H., Moreira R., Rodrigues A., Santos C.P. (2018). Finding Parameters around the Abdomen for a Vibrotactile System: Healthy and Patients with Parkinson’s Disease. J. Med. Syst..

[19] Paszkiel S. (2020). Data Acquisition Methods for Human Brain Activity. Analysis and Classification of EEG Signals for Brain-Computer Interfaces.

[20] Stoeckel L., Garrison K., Ghosh S., Wighton P., Hanlon C., Gilman J., Greer S., Turk-Browne N., DeBettencourt M., Scheinost D. (2014). Optimizing real time fMRI neurofeedback for therapeutic discovery and development. NeuroImage Clin..

[21] Thibault R.T., MacPherson A., Lifshitz M., Roth R.R., Raz A. (2018). Neurofeedback with fMRI: A critical systematic review. NeuroImage.

[22] Sulzer J., Haller S., Scharnowski F., Weiskopf N., Birbaumer N., Blefari M., Bruehl A., Cohen L.G., Decharms R., Gassert R. (2013). Real-time fMRI neurofeedback: Progress and challenges. NeuroImage.

[23] Zotev V., Krueger F., Phillips R., Alvarez R.P., Simmons W.K., Bellgowan P., Drevets W.C., Bodurka J. (2011). Self-Regulation of Amygdala Activation Using Real-Time fMRI Neurofeedback. PLoS ONE.

[24] Young K.D., Zotev V., Phillips R., Misaki M., Yuan H., Drevets W.C., Bodurka J. (2014). Real-Time fMRI Neurofeedback Training of Amygdala Activity in Patients with Major Depressive Disorder. PLoS ONE.

[25] Young K.D., Siegle G.J., Zotev V., Phillips R., Misaki M., Yuan H., Drevets W.C., Bodurka J. (2017). Randomized Clinical Trial of Real-Time fMRI Amygdala Neurofeedback for Major Depressive Disorder: Effects on Symptoms and Autobiographical Memory Recall. Am. J. Psychiatry.

[26] Young K.D., Misaki M., Harmer C.J., Victor T., Zotev V., Phillips R., Siegle G.J., Drevets W.C., Bodurka J. (2017). Real-Time fMRI Amygdala Neurofeedback Changes Positive Information Processing in Major Depressive Disorder. Biol. Psychiatry.

[27] Hwang J., Hwang W. (2011). Vibration Perception and Excitatory Direction for Haptic Devices. J. Intell. Manuf..

[28] Watanabe H., Ujike H. (2012). Comfort in observing stereoscopic images reduced by vibration stimuli. Health.

[29] Hiraba H., Inoue M., Gora K., Sato T., Nishimura S., Yamaoka M., Kumakura A., Ono S., Wakasa H., Nakayama E. (2014). Facial Vibrotactile Stimulation Activates the Parasympathetic Nervous System: Study of Salivary Secretion, Heart Rate, Pupillary Reflex, and Functional Near-Infrared Spectroscopy Activity. BioMed Res. Int..

