# Supplementary material for: Low Cost MR Compatible Haptic Stimulation with Application to fMRI Neurofeedback
Source: Brain Sci. 2020 Oct 28;10(11):790. doi: 10.3390/brainsci10110790 (PMC7692267; doi:10.3390/brainsci10110790)
Supplement: Supplementary file 1 [file brainsci-10-00790-s001.zip › Supplementary Material/Fig_S1.pdf]

Figure S1

Shopping List

Vibrating element

Any 3-5 volt DC vibrating motor - $30

(available from Amazon)

Tube to get from the vibrating element to the subject

30’ of 1” PVC tube - $15

Paracord – 100 ft - $10

Whatever will touch the subject, e.g., tennis ball, tape, and if you want it, pipe insulation - $5

(available from Hardware store, e.g. Lowe’s)

Controller

Arduino Uno - $25

Controller - $50

Adafruitmotor Shield - $20

(available from Amazon)

Software

We have used Eprime and Matlab for this
