# Supplementary figures and images for: Low Cost MR Compatible Haptic Stimulation with Application to fMRI Neurofeedback
Source: Brain Sci. 2020 Oct 28;10(11):790. doi: 10.3390/brainsci10110790 (PMC7692267; doi:10.3390/brainsci10110790)

Figure S2

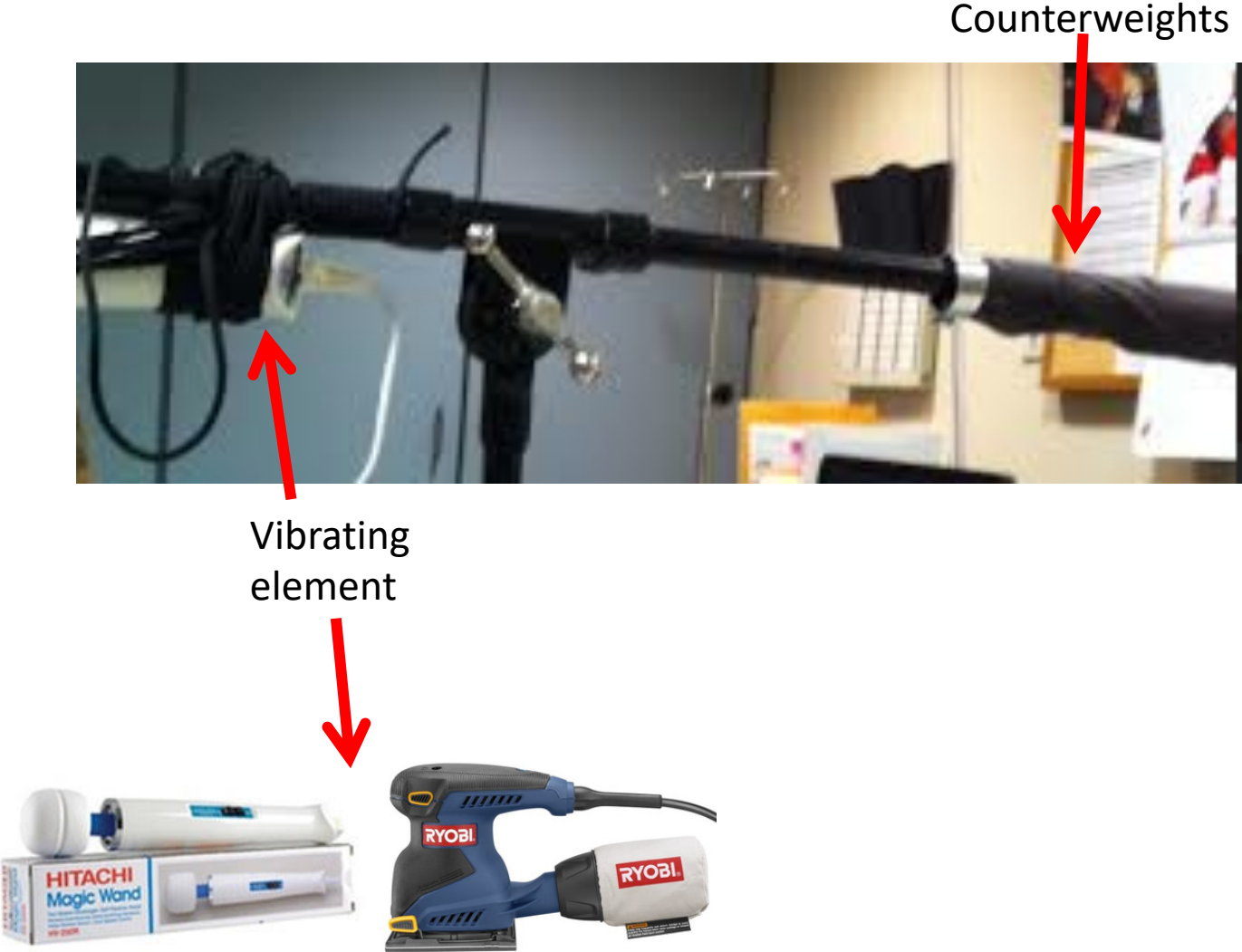

Supplement: Supplementary file 1 [file brainsci-10-00790-s001.zip › Supplementary Material/Fig_S2.pdf]
